# Editorial: Research Topic for Frontiers in Endocrinology (Obesity): Fetal Origin of Obesity and Diabetes

**DOI:** 10.3389/fendo.2022.937134

**Published:** 2022-06-30

**Authors:** Joanna H. Sliwowska, Emilyn U. Alejandro, Adam Gesing

**Affiliations:** ^1^ Laboratory of Neurobiology, Institute of Zoology, Poznań University of Life Sciences, Poznań, Poland; ^2^ Department of Integrative Biology and Physiology, University of Minnesota Medical School, Minneapolis, MN, United States; ^3^ Department of Endocrinology of Ageing, Medical University of Łódz, Łódź, Poland

**Keywords:** obesity, diabetes, fetal programming, fetal origin of adult disease, sexual dimophism

Obesity, a major risk factor for type 2 diabetes, has become a global epidemic and represents an urgent research and medical topic. Rates of overweight and obesity continue to grow both in adults and children. Importantly, according to the concept of fetal programing, the development of obesity has an origin very early in life, starting *in utero*, as a result of metabolic or nutrient stress in pregnant mothers. Moreover, the fetal origin of adult diseases theory implicates the contribution of the early environment in the womb to the development of various diseases such as cardiovascular disease and diabetes in later life. Finally, the pandemic of COVID-19 may further aggravate the obesity problem due to lockdowns, remote working/schooling, a more sedentary style of living, reduced physical activity, and increased consumption of an unhealthy diet in excess of need. Thus, obesity is also a major societal challenge, which requires a multidisciplinary approach.

This Research Topic entitled “Fetal Origin of Obesity and Diabetes” covered a variety of aspects of obesity (from basic science, through clinical studies to treatment and intervention approaches). Here we focus on the following topics: 1) fetal programming of obesity, 2) sexual dimorphic effects of obesity, and 3) interventions and treatment strategies in children.

This Research Topic consists of five original articles, one review, and one mini-review.

The first original paper by Qian Zhang et al. 1 focused on long-term effects of intrauterine energy intake on brown adipose tissue (BAT) metabolism in adulthood. To achieve this goal, high-fat diet (HFD) were given to mouse dams before and during pregnancy and lactation. The authors employed histological techniques using hematoxylin and eosin standing and DNA methylation arrays to assess the genome-wide methylation profile of BAT. They concluded that maternal HFD leads to long-lasting alterations of BAT in offspring. These changes include the activation of BAT-specific genes important for fatty acid oxidation and thermogenesis *via* DNA methylation.

In the second original paper by Annelene Govindsamy et al. 2 expression profiles of factors in the neonatal female, and male whole neonatal rat hearts were studied in response to varying dietary fat content. First, they performed immunohistochemistry for insulin receptor, glucose transporter 4 (Glut4), and forkhead box protein 1 (FoxO1) in whole hearts of neonates from dams fed with 10%, 20%, 30% or 40% high fat diet. They also examined expression levels of 84 genes, involved in the insulin signaling pathway in 10% or 40% neonates. Overall, this study shows that maternal diet may impact cardiac physiology in the offspring and highlight divergent mechanisms that are sex- -dependent.

The purpose of the third original paper by Brittany B. Rice et al. 3 was to investigate glucose tolerance and body composition to a toxicant polychlorinated biphenyls (PCB)-exposed offspring expressing or lacking nuclear factor erythroid-2-related factor 2 (Nrf2). The authors hypothesized that offspring lacking Nrf2 expression would be more susceptible to the long-term effects of perinatal PCB exposure. The whole-body Nrf2 heterozygous (Het) and Nrf2 knockout (KO) mice were exposed during gestation to vehicle or PCB_126_ and litters were cross-fostered to unexposed dams. Bodyweight, body composition, and glucose tolerance were analyzed in offspring after weaning. In this study, the authors found that: 1) In offspring born to Nrf2 dams at two months of age, PCB exposure decreased body weight of, which was associated with reduction in average lean body mass; 2) in PCB-exposed offspring born to Nrf2 dams there was no difference in average body weight; 3) after an oral glucose challenge, offspring exposed to PCB had impairments in glucose disposal and uptake. The Authors plan to focus in the future on revealing effects of maternal Nrf2 genotypic differences on offspring metabolic responses to *in utero* PCB exposure.

The fourth original paper by Brian Akhaphong et al. 4 aimed to determine how high-fat diet (HFD) challenge 4 weeks before pregnancy and during pregnancy alters the long-term metabolic outcomes in the adult male and female offspring. Studies were performed on C57BL/6J dams, which received either control or 60% kcal HFD. Glucose homeostasis was assessed in the adult offspring. It was found that: 1) Both control and HFD-dams had higher weight during pregnancy, however HFD-dams gained more weight compared to control dams; 2) There was no difference in litter size and offspring birthweight in HFD-dams and control-dams; 3) In newborns from HFD-dams there was a reduction in random blood glucose; 4) In newborns from HFD, there was no changes in islet morphology and alpha-cell fraction, but a reduction in beta-cell fraction was found; 5) In adult male offspring of HFD-dams and these on normal chow there was a comparable glucose tolerance; 6) Male offspring, which were re-challenged with HFD showed glucose intolerance transiently; 7) On the other hand, adult female offspring born of HFD-dams displayed normal glucose tolerance but had increased insulin resistance; 8) Adult female offspring of HFD-dams had increased insulin secretion in response to high-glucose treatment, but no change in beta-cell mass. It was concluded that maternal HFD at pre-conception and during gestation leads to insulin resistance in adult female offspring.

The fifth original research paper’s by Tina Linder et al. 5 goal was to study the impact of pre-pregnancy overweight on treatment modalities of Gestational Diabetes Mellitus (GDM). First, the authors investigated whether there is an association between increased pregravid Body Mass Index (BMI) with dosing of basal and rapid-acting insulin. Pregnancy outcomes were assessed from 509 women with gestational diabetes (normal weight: 201, overweight: 157, obese: 151). Second, a prospectively compiled database was analyzed to assess association between pregnancy outcomes and insulin or metformin treatments. The authors reported that: 1) There was an association between increased BMI and the need of glucose lowering medication; 2) Mothers with pregestational obesity received the highest amount of insulin; 3) Use of metformin was more often reported in patients with obesity who also required higher daily doses; 4) There was an association between maternal BMI and an increased risk of cesarean section as well as, delivering large for gestational age offspring; 5) Patients with obesity who required glucose lowering therapy had the highest birthweight percentiles. The authors concluded that treatment modalities and outcomes in GDM pregnancies are closely related to the extent of maternal BMI. Moreover, data indicate that patients with obesity required glucose-lowering medication more often and were at higher risk of adverse pregnancy outcomes. Finally, it was emphasized that there is a need to continue studies to explore the underlying pathophysiologic mechanisms. Such approach allow to optimize clinical management and individual treatment approaches.

The review paper by Emilia Grzęda et al. 6 aimed to presents the epidemiological and experimental data considering maternal undernutrition, obesity, and diabetes. The results are discussed in the context of the fetal programming with the focus on the fetal metabolic system and central nervous system (CNS). Moreover, the authors investigated the mechanism(s) underlying the sexual dimorphism of metabolic diseases both in the offspring of malnourished and over nourished mothers. It was concluded that basic science studies could provide useful tools for the prevention and treatment of these diseases in offspring. Above disused subjects are of particular relevance at the time of the pandemic and the post-COVID period when reduced physical activity and increase the consumption of unhealthy food products participate to observe rise in incidence of obesity. Finally, it was emphasized that as the womb may be more important than home, there is an urgent need to implement these strategies not only during times of pregnancy and lactation but also long before conception.

The mini-review paper by Andrzej Bartke et al. 7 focused on effects of early life hormonal interventions on alterations of the trajectory of aging. The authors employed growth hormone (GH) deficient Prop1^df^ (Ames dwarf) mice model, which is characterized by remarkably long-lived. The main finding of this group are as followed: 1) In the mutants animals GH injections between the ages of two and eight weeks normalized (“rescued”) a number of adult metabolic characteristics responsible for extended longevity; 2) Longevity in this animal model was reduced by early life GH treatment and associated with histone H3 modifications. The authors concluded that early-life interventions can modify the trajectory of mammalian aging *via* epigenetic mechanisms.

In summary, this Research Topic for Frontiers in Endocrinology (Obesity): Fetal Origin of Obesity and Diabetes presented an overview of the current advances in the aspect of obesity and diabetes research and ongoing research directions. In a variety of papers discussed above, the epidemiological perspective, medical complications, and their economic consequences to basic science have emphasized the importance of this area of research and highlighted additional works needed to fully realize effective intervention and treatment strategies. We thrive to achieve an impact on the global agenda of obesity and diabetes and support additional studies in both clinical and basic sciences to move this field more mechanistic forward.

## Author Contributions

All authors listed have made a substantial, direct, and intellectual contribution to the work and approved it for publication.

## Conflict of Interest

The authors declare that the research was conducted in the absence of any commercial or financial relationships that could be construed as a potential conflict of interest.

## Publisher’s Note

All claims expressed in this article are solely those of the authors and do not necessarily represent those of their affiliated organizations, or those of the publisher, the editors and the reviewers. Any product that may be evaluated in this article, or claim that may be made by its manufacturer, is not guaranteed or endorsed by the publisher.
